# The Impact of VEGF Inhibition on Clinical Outcomes in Patients With Advanced Non-Small Cell Lung Cancer Treated With Immunotherapy: A Retrospective Cohort Study

**DOI:** 10.3389/fonc.2021.663612

**Published:** 2021-05-28

**Authors:** Keiko Tanimura, Tadaaki Yamada, Ayaka Omura, Shinsuke Shiotsu, Nobutaka Kataoka, Takayuki Takeda, Ryusuke Taniguchi, Takahiro Yamada, Mayumi Takeuchi, Yusuke Chihara, Yoshie Morimoto, Masahiro Iwasaku, Yoshiko Kaneko, Junji Uchino, Koichi Takayama

**Affiliations:** ^1^ Department of Pulmonary Medicine, Graduate School of Medical Science, Kyoto Prefectural University of Medicine, Kyoto, Japan; ^2^ Department of Respiratory Medicine, Japanese Red Cross Kyoto Daiichi Hospital, Kyoto, Japan; ^3^ Department of Respiratory Medicine, Japanese Red Cross Kyoto Daini Hospital, Kyoto, Japan; ^4^ Department of Pulmonary Medicine, Matsushita Memorial Hospital, Osaka, Japan; ^5^ Department of Respiratory Medicine, Uji-Tokushukai Medical Center, Kyoto, Japan

**Keywords:** immune checkpoint inhibitors, immunotherapy, anti-VEGF therapy, non-small cell lung cancer, treatment response

## Abstract

**Background:**

In recent years, immune checkpoint inhibitors (ICIs) in combination with chemotherapy have increased survival in patients with advanced non-small cell lung cancer (NSCLC). Vascular endothelial growth factor (VEGF), which plays a key role in tumor angiogenesis, is an immunological modulator; therefore, it is expected that anti-VEGF therapy in combination with ICIs enhances the antitumor effect of ICIs. In the present study, we investigated the impact of VEGF inhibition on clinical outcomes of NSCLC patients, including the efficacy of ICI treatment.

**Methods:**

A total of 105 patients with advanced NSCLC who had been treated with ICIs were retrospectively analyzed to examine the relationship between the history of treatment with anti-VEGF agents and the clinical outcomes with ICI monotherapy.

**Results:**

Patients who had received anti-VEGF therapy prior to ICIs showed shortened progression-free survival of ICI treatment and a decreased overall response rate to ICI treatment. By contrast, anti-VEGF therapy after ICI treatment was associated with increased survival, especially in patients who had also received anti-VEGF therapy prior to ICI therapy.

**Conclusions:**

These retrospective observations suggest that anti-VEGF therapy prior to ICIs might be a negative predictor of response to ICIs. The sequence of anti-VEGF therapy might play a role in its ability to predict survival in NSCLC patients. Further investigation is warranted to identify the role of VEGF inhibition in altering clinical outcomes after immunotherapy.

## Introduction

Lung cancer is the leading cause of cancer-related death worldwide (1). Novel strategies for treating advanced lung cancer have progressed in recent years. One strategy has been the successful treatment of non-small cell lung cancer (NSCLC) with angiogenesis inhibitors. In addition, cancer immunotherapies, including programmed cell death protein 1 (PD-1)/programmed death ligand 1 (PD-L1) and cytotoxic T lymphocyte-associated antigen 4 (CTLA-4) checkpoint inhibitors, are being developed as promising alternative strategies for treating patients with advanced NSCLC. Several phase III clinical trials have shown improved outcomes with these immune checkpoint inhibitors (ICIs), such as prolonged survival and a more durable treatment response (2–7). However, as with chemotherapy and molecular-targeted therapy, some patients show intrinsic resistance to ICIs, and a majority of patients treated with ICIs develop acquired resistance.

The tumor microenvironment (TME), which consists of blood vessels, stromal cells, fibroblasts, signaling molecules, immune cells, and extracellular matrix components, is controlled by extracellular signals released from tumors. Vascular endothelial growth factor (VEGF), which is produced by tumor cells and surrounding stromal cells, promotes angiogenesis by binding VEGF receptor (VEGFR) on vascular endothelial cells. VEGF is also a potential modulator of the innate immune response. VEGF/VEGFR signaling promotes evasion of the antitumor immune response through multiple mechanisms, including suppression of dendritic-cell maturation (8), reducing cytotoxic T lymphocyte activity *via* production of inflammatory cytokines and upregulation of immune checkpoint expression (9, 10), activation of regulatory T cells (Tregs) (11), and accumulation of myeloid-derived suppressor cells (MDSCs) (12). High levels of pretreatment serum VEGF are associated with poor response to immunotherapy in malignant melanoma (13). Further, in a preclinical model, VEGF/VEGFR blockade restores immunosuppressive alterations caused by VEGF production, and combination of VEGF/VEGFR blockade with immunotherapy improves the antitumor immune response (14).

Bevacizumab, which is a recombinant, humanized monoclonal antibody that binds all isoforms of VEGF-A, shows favorable outcomes in combination with chemotherapy in NSCLC patients (15). Additionally, a previous study demonstrated that combination of bevacizumab with the CTLA-4 inhibitor ipilimumab enhanced infiltration of CD8+ T-cells compared to ipilimumab monotherapy in malignant melanoma (16). In recent years, some clinical study demonstrated that combination of anti-VEGF agents and ICIs prolongs survival compared to anti-VEGF alone in multiple carcinomas such as renal cell carcinoma and hepatocellular carcinoma (17, 18). The IMpower150 trial demonstrated significant improvement in survival in non-squamous NSCLC patients receiving atezolizumab, bevacizumab, carboplatin, and paclitaxel compared to patients receiving bevacizumab, carboplatin, and paclitaxel (19). On the other hand, a previous study demonstrated that prior anti-VEGF therapy was a negative predictive factor for the treatment efficacy of ICIs in metastatic renal cell carcinoma (20). However, studies in NSCLC patients who had received immunotherapy indicated a survival benefit of ICIs following chemotherapy combined with an angiogenesis inhibitor (21–23). Therefore, the impact of anti-VEGF therapy on the efficacy of immunotherapy is not fully understood in NSCLC patients. In this study, we conducted a retrospective investigation focused on the impact of VEGF inhibition on clinical outcomes in patients with advanced NSCLC treated with ICIs.

## Materials and Methods

### Patients

We retrospectively analyzed the medical records of NSCLC patients who received ICIs from February 2016 to December 2017 at five institutions in Japan: the University Hospital Kyoto Prefectural University of Medicine (Kyoto, Japan), the Japanese Red Cross Kyoto Daiichi Hospital (Kyoto, Japan), the Japanese Red Cross Kyoto Daini Hospital (Kyoto, Japan), the Matsushita Memorial Hospital (Osaka, Japan), and the Uji-Tokushukai Medical Center (Kyoto, Japan). In total, 105 patients met the following inclusion criteria: 1) histologically or cytologically proven NSCLC; 2) age ≥ 20 years; 3) administered more than two cycles of anti-PD-1 antibodies (nivolumab or pembrolizumab); and 4) received systemic chemotherapy, including cytotoxic chemotherapy or molecular-targeted therapy, prior to ICI treatment. Patients were excluded if they met any of the following criteria: 1) patients who have uncontrolled serious infections, uncontrolled cardiovascular disease, and metabolic disorders, including diabetes mellitus; 2) patients who were pregnant or nursing; and 3) patients who were regarded as unsuitable for this study by the investigators.

The study was conducted in accordance with the Declaration of Helsinki. All patients provided written or electronic informed consent. The authors are accountable for all aspects of the work in ensuring that questions related to the accuracy or integrity of any part of the work are appropriately investigated and resolved. Each hospital study protocol was approved by the ethics committee of the respective hospital. We used the Response Evaluation Criteria in Solid Tumors version 1.1 for evaluation of treatment efficacy.

### Statistical Analysis

Survival curves were calculated according to the Kaplan-Meier method. Gehan-Breslow-Wilcoxon method was used to compare survival. Overall survival (OS) was defined as the interval from the initial day of treatment to death from any cause. Progression-free survival (PFS) was defined as the interval from the initial day of treatment to disease progression or death. Categorical variables, such as the overall response rate (ORR), were compared using Fisher’s exact test. Cox proportional hazards models were used for multivariate analysis of PFS and OS, and logistic regression models were used for multivariate analysis of the ORR. For all analyses, a p-value less than 0.05 indicated statistical significance.

### Software Tools

Statistical analyses were performed using GraphPad Prism8 (GraphPad Software, San Diego, CA, USA) and EZR statistical software version 1.50 (Saitama Medical Center, Jichi Medical University, Saitama, Japan), which is a graphical user interface for R (The R Foundation for Statistical Computing, Vienna, Austria). More precisely, it is a modified version of R commander designed to add statistical functions frequently used in biostatistics.

## Results

### Patient Characteristics

**Figure 1:**
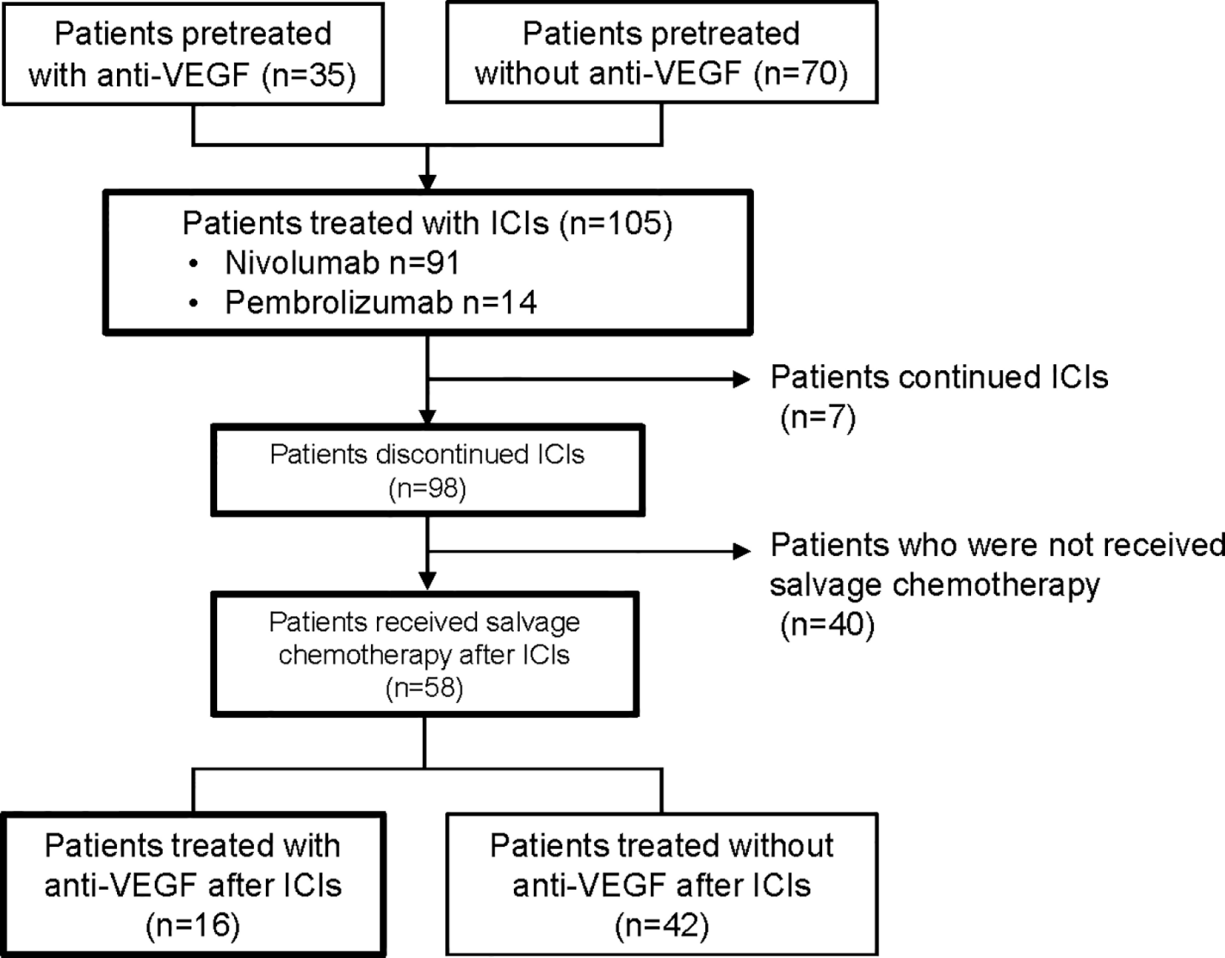
Flow diagram of patients included in the analysis. VEGF, vascular endothelial growth factor; ICI, immune checkpoint inhibitor.

The data cutoff date for the survival analysis was May 1, 2020. The median duration of follow-up for surviving participants was 36.8 months (range, 4.1-51.6 months). Of the 105 NSCLC patients treated with ICIs, 35 (33%) were treated with anti-VEGF agents before ICIs (pre-anti-VEGF) and 70 (67%) patients were not treated with anti-VEGF agents before ICIs (no pre-anti-VEGF) (**Figure 1**). The characteristics of the two groups are listed in **Table 1**. Non-squamous cell carcinoma was more common in the pre-anti-VEGF group than in the no pre-anti-VEGF group, and radiation therapy was less common in the pre-anti-VEGF group. Other factors, including the occurrence of immune related adverse events and peripheral blood findings, did not differ between the two groups (**Table 1**, **Supplementary Table 1**).

**Table 1:** Characteristics of patients included in this study.

	Pre-anti-VEGF	No pre-anti-VEGF	*p value*
	(n=35)	(n=70)	
**Characteristics at Start of ICI**			
Age-median (range), years Sex-n. (%)	69 (41–83)	71 (46-83)	0.104
Male	21 (60)	49 (70)	0.381
Female	14 (40)	21 (30)	
ECOG PS≥2 -2. (%)	3 (8.6)	14 (20)	0.167
Smoking history-2. (%)			
Current or former	26 (74.3)	54 (77.1)	0.81
Never	9 (25. 7)	16 (22.9)	
Histology-2. (%)			
Sq.	5.7 (26)	32 (45.7)	<0.001
Non-Sq.	94.3 (74)	38 (54.3)	
Driver oncogen-n. (%)			
With	28 (80)	47 (67.1)	0.069
Without	6 (17.1)	10 (14.3)	
Unknown	1 (2.9)	13 (18.6)	
PD-L 1 expression-n			
0%	4 (11.4)	3 (4.2)	0.347
<50%	5 (14.3)	12 (17.1)	
≥50%	5 (14.3)	14 (20)	
Unknown	21 (60)	41 (58.6)	
Corticosteroids and/or immunosuppressive agents -n. (%)	5 (14.3)	6 (8.6)	0.5
Autoimmune disease -n.(%)	3 (8.6)	3 (4.3)	0.398
ANC -(mean±SD, cells/rnrn^3^)	4138 ± 1857	5095 ± 3014	0.088
ALC -(mean±SD, cells/rnrn^3^)	1275 ± 518	1236 ± 573	0.23
AMC -(mean±SD, cells/rnrn^3^)	536 ± 260	544 ± 301	0.89
**Treatment history prior ICI therapy**			
Platinum-based chemotherapy -n. (%) Anti-angiogenic –n. (%)	34 (97.1)	66 (94.3)	0.663
Bevacizurnab	31 (88.6)		
Ramucirumab	9 (25. 7)		
History of radiation -n.(%)	14 (40)	44 (62.9)	0.037
Information ofiCI therapy			
**Information of ICI therapy**			
Treatment line ofiCI-median (range) ICI-n.(%)	3 (2-8)	3 (2-13)	0.346
Nivolumab	30 (85.7)	61 (87.1)	1
Pembrolimmab	5 (14.3)	9 (12.9)	
irAE	12 (34.3)	30 (42.9)	0.527

VEGF, vascular endothelial growth factor; ICI, immune checkpoint inhibitor, ECOG PS, Eastern Cooperative Oncology Group performance status; Sq. squamous cell carcinoma; PD-L1, programmed death ligand 1; ANC, absolute neutrophil count; ALC, absolute lymphocyte count; AMC, absolute monicyte count.

### Efficacy and Survival Analysis for ICIs in Patients Previously Treated With Anti-VEGF Therapy

**Figure 2:**
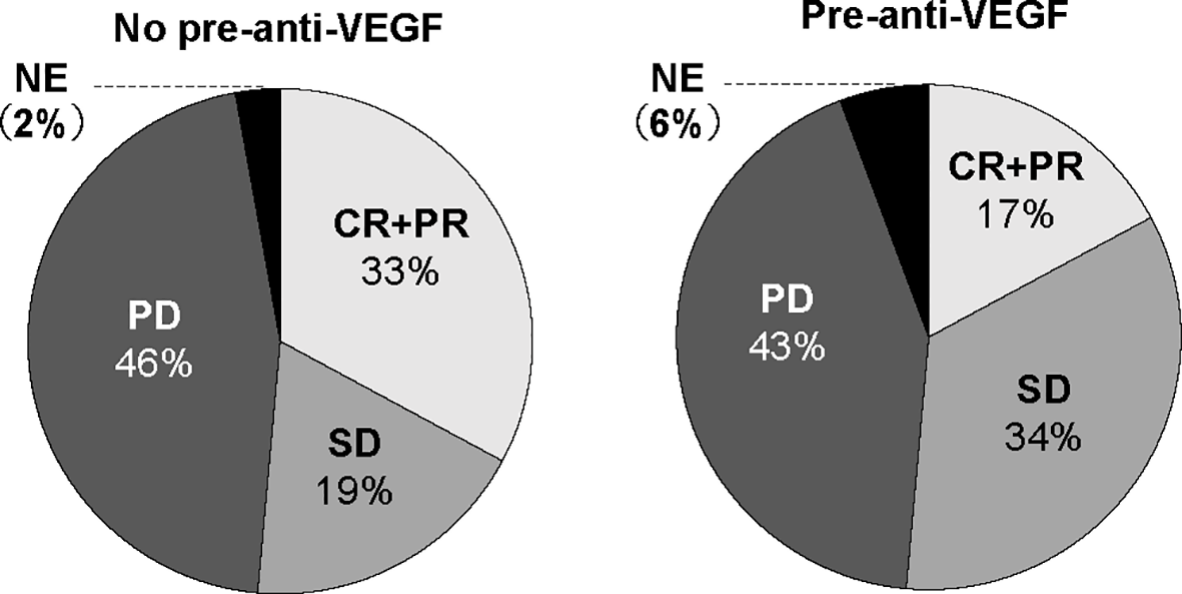
ORR to ICIs according to anti-VEGF treatment prior to ICI treatment. ORR, overall response rate; ICI, immune checkpoint inhibitor; VEGF, vascular endothelial growth factor.

**Figure 3:**
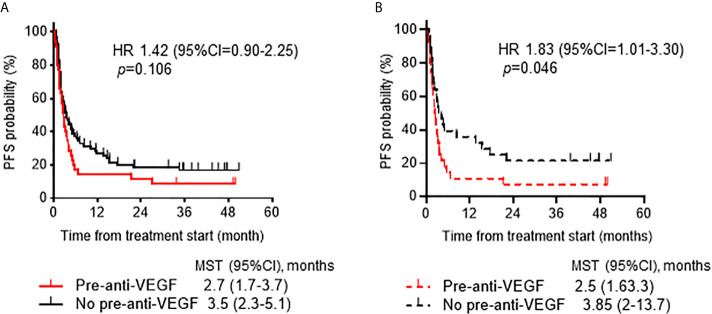
Progression-free survival ICIs according to anti-VEGF treatment prior to ICI treatment. **(A)** Kaplan-Meier analysis of PFS after ICI treatment according to history of treatment with anti-VEGF agents in the full cohort. **(B)** Kaplan-Meier analysis of PFS in the propensity score-matched cohort (1:1, n=28 /group). ICI, immune checkpoint inhibitor; VEGF, vascular endothelial growth factor; PFS, progression-free survival.

To investigate prognostic factors related to clinical outcomes, we conducted univariate and multivariate analyses for ORR, PFS and OS. The ORR of ICI treatment was significantly lower in the pre-anti-VEGF group than that in the no pre-anti-VEGF group in univariate analysis (17% and 33%, respectively; p = 0.014) (**Figure 2**). Multivariate analysis showed that a treatment history of anti-VEGF agents was independently associated with a poor response to ICI treatment [OR: odds ratio 0.30 (95% CI: 0.09-0.96), p = 0.043] (**Table 2**). In the univariate analysis of PFS and OS, patients whose Eastern Cooperative Oncology Group-performance status (ECOG-PS) scores were ≥2 had significantly poorer PFS and OS than other patients (p = 0.039 and p < 0.001, respectively) (**Supplementary Table 2**). Multivariate analysis revealed that worse ECOG-PS and a treatment history of anti-VEGF agents were independently associated with poor PFS after immunotherapy [HR 2.26 (95% CI: 1.20-4.29), p = 0.012 and HR 1.83 (95% CI: 1.05-3.20), p = 0.033, respectively] (**Table 2**). By contrast, a treatment history of anti-VEGF agents was not significantly associated with OS [HR 1.47 (95% CI: 0.76-2.82), p = 0.249] (**Table 2**). Furthermore, propensity score matched analysis was performed to balance the characteristics between groups with or without anti-VEGF therapeutic history. After matching, the mean propensity scores for pre-anti-VEGF and no-anti-VEGF were 0.444 ± 0.162 and 0.444 ± 0.162, respectively (p = 1) (**Supplemnetary Table 3**). Pre-anti-VEGF group showed marginally shorten PFS of ICI treatment in the full cohort, however, this tendency was emphasized in the property score matched cohort (**Figure 3**).

**Table 2:** Multivariate analysis of ORR, PFS and OS.

	ORR		PFS		OS	
	OR (95%CI)	*P* value	HR (95%CI)	*P * value	HR (95%CI)	*P* value
Age, < 70y	Reference		Reference		Reference	
≥70y	1.12 (0.45-2.79)	0.803	0.77 (0.49-1.19)	0.237	1.14 (0.70-1.86)	0.605
Gender						
Female	Reference		Reference		Reference	
Male	0.60 (0.17-2.09)	0.42	1.06 (0.58-1.92)	0.86	1.23 (0.59-2.57)	0.576
ECOG PS						
<2	Reference		Reference		Reference	
≥2	0.51 (0.13-1.97)	0.328	2.26 (1.20-4.29)	0.012	4.50 (2.22-9.11)	<0.001
Smoking history						
Never	Reference		Reference		Reference	
Current or former	1.60 (0.39-6.66)	0.516	0.83 (0.42-1.65)	0.599	1.03 (0.46-2.31)	0.948
Histology						
Sq.	Reference		Reference		Reference	
Non-Sq.	1.71 (0.60-4.91)	0.317	0.67 (0.39-1.17)	0.162	0.54 (0.29-1.02)	0.057
Corticosteroid and/or inunnosuppressive agents						
	2.01 (0.46-8.69)	0.352	1.13 (0.57-2.23)	0.734	1.24 (0.58-2.65)	0.584
History of radiation	1.00 (0.39-2.55)	0.993	0.76 (0.49-1.18)	0.228	0.98 (0.59-1.63)	0.934
History of anti- VEGF	0.30 (0.09-0.96)	0.043	1.83 (1.05-3.20)	0.033	1.47 (0.76-2.82)	0.249

PFS, progression free survival; OS, overall survivat ORR, overall response rate; HR, hazard ratio; OR, Odds Ratio; CI, confidence intervat ECOG PS, Eastern Cooperative Oncology Group performance status; Sq. squamous cell carcinoma; VEGF,vascular endothelial growth factor.

### Effect of Sequence of Anti-VEGF Agents With Regard to ICI Treatment on Patient Outcomes

**Figure 4:**
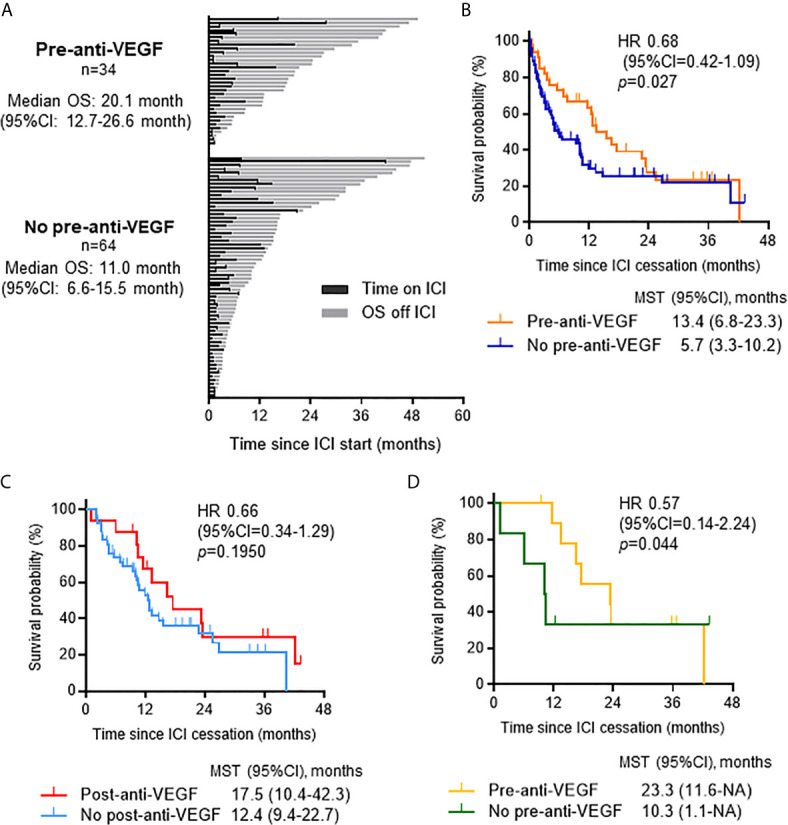
Impact of anti-VEGF therapy on survival when administered pre- or post-ICI therapy. **(A)** Swimmer plot for patients who discontinued ICIs (n = 98) categorized by whether they received anti-VEGF therapy prior to ICIs. **(B)** Kaplan-Meier analysis of survival from ICI cessation according to history of treatment with anti-VEGF agents. Gehan-Breslow-Wilcoxon method was used to compare survival. **(C)** Kaplan-Meier analysis of survival from ICI cessation in patients who received salvage chemotherapy after ICIs (n = 58) according to post-ICI anti-VEGF treatment. **(D)** Kaplan-Meier analysis of survival from ICI cessation in patients who received post-ICI anti-VEGF therapy (n = 16) according to therapeutic history of anti-VEGF agents prior to ICIs. Gehan-Breslow-Wilcoxon method was used to compare survival. VEGF, vascular endothelial growth factor; OS, overall survival; CI, confidence interval; ICI, immune checkpoint inhibitor; HR, hazard ratio; MST, median survival time.

To further reveal the impact of VEGF inhibition on patient outcomes, we examined the effect of the sequence of anti-VEGF therapy related to ICI treatment. Among the 98 patients who discontinued ICI treatment, previous treatment with anti-VEGF agents (n = 34) showed a trend toward prolonged OS compared to no previous treatment with anti-VEGF agents (n = 64) [median OS 20.1 months (95% CI 12.7-26.6) and 11.0 months (95% CI 6.6-15.5), respectively; *p* = 0.216] and post-ICI survival time [HR: 0.68 (95% CI: 0.42-1.09), *p* = 0.027] (**Figures 4A, B**). Patients who received anti-VEGF agents after ICIs tended to have longer post-ICI survival than those who did not receive anti-VEGF therapy after ICIs [HR 0.66 (95% CI 0.34-1.29), *p* = 0.195] (**Figure 4C**). Furthermore, a therapeutic history of anti-VEGF therapy prior to ICIs was associated with a favorable prognosis after ICIs among patients who received anti-VEGF therapy after ICIs [HR: 0.57 (95% CI: 0.14-2.24), *p* = 0.044) (**Figure 4D**).

## Discussion

This retrospective study revealed the impact of the sequence of anti-VEGF inhibition on clinical outcomes related to ICI treatment in NSCLC patients. Anti-VEGF therapy prior to ICIs was shown to be a negative predictor of response to ICI treatment. The sequence of anti-VEGF therapy might play a role in its ability to predict survival in NSCLC patients. Thus, it is important to establish a therapeutic strategy that maximizes the therapeutic effect of ICIs and anti-VEGF therapy.

Currently, immunotherapy is widely used in multiple cancers including lung cancer, and the multiple biomarkers related to the efficacy of ICI treatment have recently emerged, such as PD-L1 expression in tumor and/or immune cells and tumor mutation burden (5, 24, 25). Previous studies have reported that the profile of immune cells in the TME or circulating peripheral blood might be a predictor of the therapeutic effect of immunotherapy (26–30). Tumor-infiltrating CD8+T cells, which exert an effector function in the TME, are associated with favorable clinical outcomes in patients receiving immunotherapy, whereas accumulation of immunosuppressive cells, such as MDSCs and Tregs, which are major mediators of immune tolerance, correlates with poor prognosis (26–28). These immune-related cells are regulated by molecules secreted by TME components and negatively regulate the function of effector T-cells. They can also inhibit the differentiation and maturation of dendritic cells (29, 30).

VEGF, a molecule secreted by tumor and stromal cells, plays pivotal roles in the TME. The TME is characterized by hypoperfusion, hypoxia, acidity, and high interstitial fluid pressure through tumor vascularization, which can lead to tumor growth and drug resistance. Anti-VEGF agents reverse these changes; therefore, combination treatment with anti-VEGF agents can be expected to enhance the therapeutic effect of ICIs. Previous studies have identified mechanisms of intrinsic resistance to immunotherapy related to previous treatment with anti-angiogenic therapies (31–33). One such mechanism is that anti-VEGF therapy induces compensatory upregulation of VEGF-independent angiogenic factors, which contribute to the production of an immunosuppressive TME. VEGF/VEGFR2 is a major signaling axis driving tumor angiogenesis, and blockade of this pathway induces upregulation of VEGF-independent compensatory pro-angiogenic factors, including fibroblast growth factor (FGF) 2, platelet-derived growth factor, and angiopoietin-1 and 2 (31). In preclinical models, bone marrow-derived fibrocyte-like cells, which are induced by anti-VEGF therapy, contribute to resistance to anti-VEGF therapy by upregulating FGF2, and the activated FGF/FGF receptor pathway is associated with non-T-cell inflamed tumors that show resistance to immunotherapy (32, 33). These previous studies suggest that multiple pro-angiogenic factors might be upregulated after VEGF inhibition, leading to attenuation of the antitumor immune response following ICI treatment. Another potential mechanism is that VEGF/VEGFR2 signaling, which is suppressed by anti-VEGF therapy, is immediately reactivated after withdrawal of anti-VEGF agents and returns to baseline, thereby inducing rapid revascularization (34).

In this study, anti-VEGF treatment prior to ICI treatment was associated with poor response to ICIs; however, salvage chemotherapy with anti-VEGF agents after ICI treatment increased survival. These observations support this second hypothesis and suggest that blockade of VEGF/VEGFR signaling might be reversible and that it is essential for patient survival after ICIs and prior anti-VEGF therapy. Additionally, pharmacodynamic analysis revealed that anti-PD-1 antibody remained bound even 2 months after infusion (35). Therefore, previous ICI treatment may improve the effect of any subsequent therapy, including anti-VEGF therapy. Further investigation is warranted to determine the optimal treatment strategy and sequence for ICIs and anti-VEGF therapy.

This study has several limitations. First, this was a retrospective study, so the cohort had a limited sample size and may be subject to patient selection bias. Second, additional analyses using peripheral blood or tumor samples, such as changes in secreted angiogenic factors and phenotypes of immune systems, were not performed, and the precise mechanisms of resistance to ICI were not elucidated. Third, PD-L1 expression, which is a predictive biomarker of ICI response, was measured in only some cases. Finally, the study does not examine the current standard regimens for advanced NSCLC: platinum-based chemotherapy combined with ICIs and angiogenesis inhibitors. Optimal treatment strategies that maximize the therapeutic effect of ICIs and anti-VEGF therapies should be investigated in future studies.

In conclusion, our study demonstrated poorer response to immunotherapy in patients previously treated with anti-VEGF agents than in patients not previously treated with anti-VEGF agents. However, salvage chemotherapy with anti-VEGF agents after ICI failure was associated with prolonged survival. These results suggest that the interventional sequence and maintenance of VEGF inhibition may be important and contribute to clinical outcomes in patients with advanced NSCLC. Further investigation is needed to determine the optimal treatment strategy for immunotherapy and anti-VEGF therapy.

## Data Availability Statement

The original contributions presented in the study are included in the article/**Supplementary Material**. Further inquiries can be directed to the corresponding author.

## Ethics Statement

The studies involving human participants were reviewed and approved by Kyoto Prefectural University of Medicine. The patients/participants provided their written informed consent to participate in this study.

## Author Contributions

Conception and design: KeT and TadY. Administrative support: TakY and KoT. Provision of study materials or patients: SS, TT, TakY, YC, YM, MI, YK, and JU. Collection and assembly of data: KeT, AO, NK, RT, and MT. Data analysis and interpretation: KeT and TadY. All authors contributed to the article and approved the submitted version.

## Funding

This work was supported by research grants from JSPS KAKENHI [Grant Numbers 20K22820 (to KeT) and 19K08608 (to TadY)].

## Conflict of Interest

TadY received commercial research grants from Pfizer, Ono Pharmaceutical, Chugai Pharmaceutical, and Takeda Pharmaceutical Company Limited. JU received research grants from Eli Lilly Japan K.K., AstraZeneca K.K., and Boehringer Ingelheim Japan. KoT received research grants from Chugai-Roche and Ono Pharmaceutical and personal fees from AstraZeneca, Chugai-Roche, MSD-Merck, Eli Lilly, Boehringer Ingelheim, and Daiichi Sankyo.

The remaining authors declare that the research was conducted in the absence of any commercial or financial relationships that could be construed as a potential conflict of interest.

## Abbreviations

PD-1, programmed cell death 1; PD-L1, programmed death ligand 1; CTLA-4, cytotoxic T lymphocyte-associated antigen 4; NSCLC, non-small cell lung cancer; ICI, immune checkpoint inhibitor; TME, tumor microenvironment; VEGF: vascular endothelial growth factor; VEGFR, vascular endothelial growth factor receptor; Treg, regulator T cell; MDSC, myeloid-derived suppressor cell; OS, overall survival; PFS, progression-free survival; ORR, overall response rate; ECOG-PS, Eastern Cooperative Oncology Group-performance status; CI, confidence interval; HR, hazard ratio; FGF, fibroblast growth factor.
